# Advances in cellular and molecular pathways of salivary gland damage in Sjögren’s syndrome

**DOI:** 10.3389/fimmu.2024.1405126

**Published:** 2024-07-10

**Authors:** Wenxia Qi, Jiexiang Tian, Gang Wang, Yanfeng Yan, Tao Wang, Yong Wei, Zhandong Wang, Guohua Zhang, Yuanyuan Zhang, Jia Wang

**Affiliations:** ^1^ Gansu University of Traditional Chinese Medicine, College of Integrative Medicine, Lanzhou, China; ^2^ Affiliated Hospital of Gansu University of Traditional Chinese Medicine, Department of Rheumatology and Orthopedics, Lanzhou, China; ^3^ Fourth Affiliated Hospital of Gansu University of Traditional Chinese Medicine, Department of Respiratory and Critical Care Medicine, Lanzhou, China

**Keywords:** Sjögren’s syndrome, salivary gland damage, pathomechanisms, cellular, molecular biology

## Abstract

Sjögren’s Syndrome (SS) is an autoimmune disorder characterized by dysfunction of exocrine glands. Primarily affected are the salivary glands, which exhibit the most frequent pathological changes. The pathogenesis involves susceptibility genes, non-genetic factors such as infections, immune cells-including T and B cells, macrophage, dendritic cells, and salivary gland epithelial cells. Inflammatory mediators such as autoantibodies, cytokines, and chemokines also play a critical role. Key signaling pathways activated include IFN, TLR, BAFF/BAFF-R, PI3K/Akt/mTOR, among others. Comprehensive understanding of these mechanisms is crucial for developing targeted therapeutic interventions. Thus, this study explores the cellular and molecular mechanisms underlying SS-related salivary gland damage, aiming to propose novel targeted therapeutic approaches.

## Introduction

**Figure 1:**
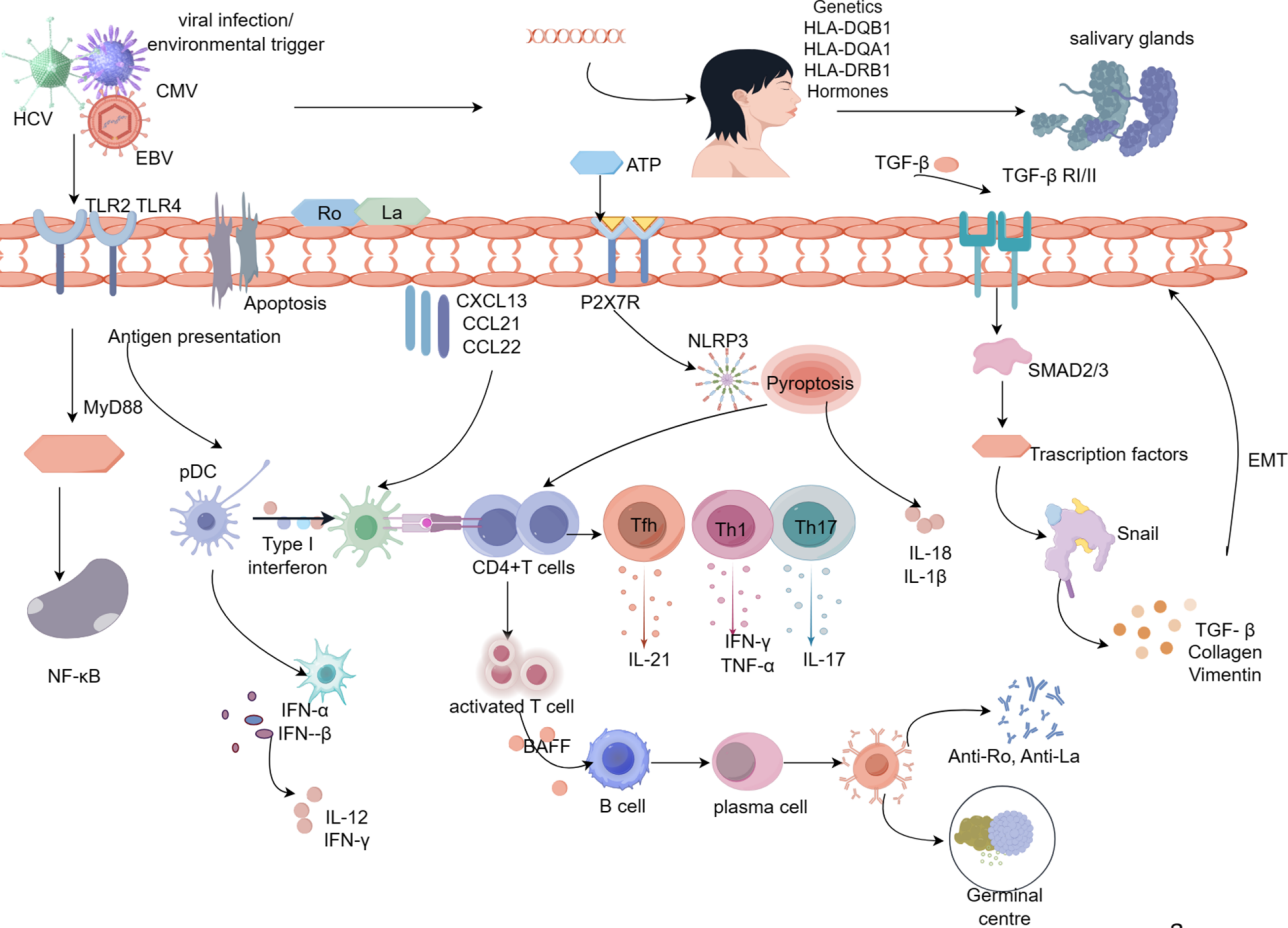
Pathologic mechanisms of salivary gland damage in Sjögren’s syndrome. Viral infection, hormonal imbalance, genetic susceptibility, and apoptosis lead to activation of SGECs, resulting in the release of the intracellular antigens Ro/La onto the surface of SGECs. Ro and La proteins activate immune cells on the extramembrane surface via TLR, and CD4^+^ T lymphocytes are activated via MHC-II molecules, which are expressed on the surface of SGECs.Activated CD4^+^T cells differentiate into Th17, Th1, and Tfh cells in response to inflammatory cytokines.SGECs interact with CD4^+^ T cells through inflammatory cytokines, repressors, leading to the production of pro-inflammatory cytokines (IL-21, IL-17, IFN-γ, TNF-α).With the involvement of BAFF, CD4^+^ T cells activate B lymphocytes, ultimately leading to the formation of autoantibodies and germinal centers. Abnormal upregulation of TGF-β1 in SGECs leads to morphological and functional mesenchymal changes in SGECs through activation of the TGF-β1/SMAD/Snail signaling pathway, which contributes to the process of SG fibrosis. Prolonged inflammatory stimulation results in a marked elevation of ATP, which activates P2X7R, and activated P2X7R allows NLRP3 to enter the cytoplasm thereby activating the NLRP3 inflammatory vesicles. activation of NLRP3 generates activated Caspase-1, which in turn cleaves the GSDMD, which punches holes in the cytosol at the N-terminal end, releasing mature IL-1β, IL-18, and thus activating the initial T cells, the differentiate into Th1 and Th17, which further secrete inflammatory cytokines.

SS is a prevalent autoimmune disorder primarily characterized by lymphocytic infiltration of exocrine glands, leading to dryness in the mouth and eyes (1). Additionally, about 30–40% of patients experience systemic complications affecting the kidneys, lungs, nervous system, and other organs (2–4). The primary target organs in SS are the exocrine glands, particularly the salivary glands (SG). The reduction in saliva production is closely linked to continuous immune cells infiltration around the salivary gland epithelial cells (SGECs) and progressive destruction of glandular structures. SG histopathological changes are predominantly marked by extensive lymphocytic infiltration into the glandular interstitium, significant SGECs destruction and atrophy, and alterations in the glandular ducts, including dilatation and narrowing, culminating in SG damage (5, 6). This chronic inflammation eventually leads to irreversible fibrosis in SGECs, characterized by an accumulation of excessive connective tissue and extracellular matrix, ultimately resulting in the loss of SG function (7). The pathogenesis of SS is intricately linked to immune system abnormalities, which involve the secretion of pro-inflammatory mediators through both innate and adaptive immune responses. Various pathological mechanisms include activation of the type I interferon (IFN) system, antigen presentation, and T and B lymphocyte activation, along with the formation of ectopic lymphoid structures (ELS) (8). Viral and genetic factors initiate the immune response, leading to lymphocytic infiltration in the SG and the release of numerous pro-inflammatory mediators, such as interleukins (IL), IFN, and members of the tumor necrosis factor (TNF) super family, which further damage the SG (9). Additionally, SGECs play a critical role in local immune responses and disease progression in SG, primarily through their secretion of cytokines and chemokines that activate and recruit T and B cells, as well as circulating peripheral immune cells (10). Inflammatory cytokines can activate specific pathways within SGECs by binding to lymphocytes and surface receptors on SGECs, further promoting the production of inflammatory factors and chemokines, thereby exacerbating the inflammatory response (11). A thorough understanding of the cellular and molecular mechanisms underlying SG damage in SS and strategies to maximize the preservation of SG secretory function are crucial for enhancing the quality of life for patients. Details can be found in **Table 1** and **Figure 1**.

**Table 1:** Common clinical symptoms, signs, and related diseases involved in SS.

Area/system involved in the body	clinical symptom/signs/associated diseases of accumulation	references
Oral cavity	dry mouth; Rampant tooth decay; Salivary gland inflammation; ParotitisAtrophy of the lingual papillae	(12, 13)
Eye	Keratoconjunctivitis sicca; Lacking tears; Lacrimal gland Enlargement; Eyelid swelling; Corneal ulcer	(13, 14)
Mucous membranes of the skin	Vasculitis; Scattered Purpura; Nodular erythema; Raynaud’s phenomenon; Cryoglobulinemia;	(13, 15)
Musculoskeletal	Joints Arthritis; Synovitis;Primary fibromyalgia;	(16, 17)
Kidney	renal distal tubular acidosis; diabetes insipidus; Gitelman syndrome;tubulointerstitial nephritis;	(18, 19)
Respiration	Intersitial Lung Disease;airway disease	(20, 21)
Digestiy feature	Esophageal mucosal atrophy; atrophic gastritis; subclinical pancreatitis	(22, 23)
Nervous system	Hemiplegia; Myelitis; Meningitis	(24, 25)
Blood system	Leukopenia/thrombocytopenia; non-Hodgkin lymphoma	(26)

## Structural changes in salivary gland pathology in SS

In the human body, the SG encompasses the parotid, submandibular, and sublingual glands, along with numerous minor glands distributed throughout the oral mucosa. The structural composition of the SG primarily includes SGECs, immune cells, and stromal cells. The alveolar and ductal cells, which are key constituents of the SGECs, play vital roles in the gland. Salivary secretion is facilitated by the contraction of myoepithelial cells and is further regulated by parasympathetic neurotransmitters such as muscarinic agents. The secreted saliva initially travels through the intercalary ducts from smaller secretory ducts and ultimately reaches the oral cavity via larger excretory ducts (27, 28). Characteristic pathological features of SG in SS patients include extensive infiltration by T and B lymphocytes around the ducts, accompanied by atrophy and a decrease in vesicular cells (29). During the early and middle stages of the disease, there is an increase in foci infiltrated by immune cells, particularly B lymphocytes, around the ductal cells, leading to the formation of lymphoid tissue germinal centers (GC) within the SG and subsequent autoantibody production. Chronic inflammatory stimulation and injury result in a marked decline in follicular and ductal cells populations, which are gradually replaced by fibrous and adipose tissue (30, 31). Electron microscopy has revealed degeneration and atrophy of SGECs, fatty infiltration within the cytoplasm, and swelling of intracellular organelles—pathological alterations that significantly diminish the secretory function of the SG (32). SGECs fibrosis is now acknowledged as a significant pathological hallmark of SS (33). Moreover, lymphoepithelial lesions, characterized by ductal basal cells and infiltrating lymphocytes predominantly surrounded by B cells, are also notable. These lesions arise when basal cells, spared from lymphocyte attacks, proliferate, leading to lymphoepithelial hyperplasia and ultimately the development of lymphoepithelial lesion (34).

## Genetic and environmental nuclear factors

Research has demonstrated that genetic factors are a significant risk factor for the development of SS. Two major genome-wide association studies (GWAS) conducted in 2013 identified risk genes in Han Chinese and European populations, revealing that many of these genes are involved in both innate and adaptive immune responses (35, 36). These studies highlighted a strong correlation between human leukocyte antigen (HLA) loci and SS, particularly noting a higher susceptibility associated with allelic variants such as HLA-DQB1*0201, HLA-DQA1*0501, and HLA-DQB1*0301 (DR3) (36). In the Chinese Han population, notable susceptibility loci include TNFAIP3 and GTF2I, which are largely related to HLA genes and the type I interferon (IFN) pathway (37, 38). While the number of GWAS focusing on SS is growing, the discovery of genetic variants with genome-wide significance remains limited compared to other autoimmune diseases (39). Epigenetics, which influences gene expression without altering the DNA sequence, primarily involves histone modification, non-coding RNA (ncRNA) activity, and DNA methylation. ncRNAs, which do not encode proteins, can form complex regulatory networks and play critical roles in bodily regulation. Abnormal expressions and functions of microRNA (miRNA), such as miR-146a, miR-155, and miR-21, can disrupt immune system functions and influence the initiation and progression of SS. Notably, miRNAs expressed in SS patients, like miR-146a/b and miR-30b-5p, may negatively regulate the B-cells activating factor (BAFF), crucial for B-cells maturation, survival, and proliferation, and pivotal in triggering SS (40). DNA methylation is also critical in SS; one study identified distinct methylation sites and regions in the whole blood of SS patients compared to healthy individuals, particularly within genes regulating type I IFN (41). Viral infections are central to the pathogenesis of SS and are considered potential triggers for initiating the autoimmune process. Viruses activate antigen-presenting cells (APCs) and autoreactive naïve T cells (42). SG serve as key sites for latent viral infections, making viral presence a major environmental factor in initiating innate immunity (43). The primary viruses implicated in SS include Epstein-Barr virus (EBV) (44), human herpesvirus (HHV) (45), human T-lymphotropic virus type 1 (HTLV), chronic hepatitis C virus (HCV) (46), and cytomegalovirus (CMV) (47). EBV is particularly significant in SS pathogenesis, capable of infecting SGECs by direct fusion with the plasma membrane or infecting B cells via the gP350/220 protein. It evades immune detection through strategies like molecular mimicry and can persist long-term within the host (48). Furthermore, EBV can exacerbate the autoimmune response by prompting the immune system to attack self-antigens through epitope spreading and bystander activation (49).

## Common cells types and pathologic mechanisms in SS

### T cells

#### Th1 and Th2 cells

Numerous studies have demonstrated that the immune response in SG affected by SS is predominantly mediated by Type 1 T helper cells (Th1) (50, 51). Elevated levels of Th1-related cytokines such as IFN-γ, TNF-α, and C-X-C motif chemokine receptor 3 (CXCR3) have been observed in the saliva of SS patients compared to those with normal SG function (52, 53), suggesting that increased lymphocyte infiltration is associated with an upregulation of Th1 cytokines. Specifically, IFN-γ plays a crucial role in SG dysfunction in the SS Ro60 humanized model (54). This cytokine is believed to contribute to SG dysfunction by disrupting tight junction structures within SS patients’ SG (55). Similarly, TNF-α, another pivotal Th1 cytokine, shows significantly higher expression in the saliva and SG of SS patients (56). Like IFN-γ, increased TNF-α levels compromise the integrity of tight junctions in salivary gland epithelial cells (SGECs) and diminish the population of vesicular cells, thereby impairing the SG’s secretory function (57). Amphiregulin (AREG), a critical growth factor, plays a vital role in the secretion of pro-inflammatory cytokines within the SG of SS patients. TNF-α has been shown to induce AREG secretion, which leads to damage in SG follicular and ductal cells (58). Additionally, TNF-α can trigger apoptosis and enhance the transcriptional expression of intercellular adhesion molecule-1 (ICAM-1) and macrophage inflammatory protein-3 (CCL20) in SGECs (59). Th2 cells, on the other hand, facilitate B cells responses through their cytokines (60). Th2 molecules have been identified in the intra-GC regions of SS patients, indicating that Th2 cells may play a role in regulating the initial B cells response (61).

#### Th17 and Treg cells

Th17 cells are a subtype of pro-inflammatory CD4^+^T cells that play a crucial role in initiating and advancing SS (62). Regulatory T cells (Treg), on the other hand, serve an inhibitory function by releasing soluble mediators or through direct cells-to-cells contact, thus maintaining the dynamic equilibrium of human immunity (63). Th17 cells produce a range of cytokines (IL-17, IL-17A, TNF-α), which collectively mediate the inflammatory response in the SG (64). Research has shown that the balance between Treg and TGF-β levels in the SG of early-stage SS model mice is significantly disrupted compared to normal controls due to the overactivation of Th17 and IL-17 cells (65). In SS patients, the expression of the transcriptional coactivator with PDZ-binding motif (TAZ) is markedly elevated (66). TAZ is known to promote Th17 differentiation and inhibit Treg development, leading to a disruption in immune homeostasis, which supports the involvement of Th17/Treg imbalance in SG damage. Clinical studies have confirmed that Th17 cells are significantly increased in the SG of SS patients compared to healthy individuals (67) and are positively correlated with the severity of the pathology (68). IL-17 induces the secretion of pro-inflammatory factors (TNF-α, IL-6, and IL-1β) and attracts immune cells to the SG by promoting the release of IL-8, C-X-C motif chemokine ligand 9, and C-C chemokine receptor type 3 (69). Furthermore, elevated IL-17A expression in the SG of SS patients has been linked to increased disease severity (70). Another Th17 cytokine, IL-22, also contributes to SG pathology. Firstly, IL-22 recruits B cells and lymphoid aggregates, facilitating the formation of ectopic lymphoid structures (ELS). Secondly, IL-22 enhances autoantibody production by stimulating the expression of cytokines such as CXCL12 and CXCL13 (71).

#### Tfh and Thr cells

Follicular helper T cells (Tfh) are a subset of CD4^+^T cells that enhance B cells differentiation and antibody production by expressing the chemokine receptor CXCR5, which interacts with the chemokine ligand CXCL13 on the surface of B lymphocytes in the germinal center (72). Elevated levels of CXCL13 have been detected in the SG of patients with SS (73). Some studies have linked increased serum concentrations of CXCL13 with abnormal B cells parameters and even lymphomagenesis (74). Tfh cells are abundantly present in the SG of SS patients and are positively correlated with disease activity (75). Moreover, Tfh cells are closely associated with the formation of ELS, and increased numbers of activated Tfh cells have been observed in the SG during the development of ectopic GC (61). IL-21 is a key effector cytokine of Tfh cells (76). Devangi and colleagues observed that IL-21 induces apoptosis in B cells and also inhibits growth and induces apoptosis in lymphoma cells, suggesting that IL-21 may also promote apoptosis in SGECs (77). Follicular regulatory T cells (Tfr), derived from FOXP3^+^ Treg cells, represent a distinct class of regulatory T cells. Tfr cells inhibit the function of Tfh and B cells within germinal centers, primarily by negatively regulating Tfh cells, thereby reducing or inhibiting autoantibody production (78). The balance between Tfh and Tfr cells is crucial for maintaining normal immune function. Research using a mouse model of Sjögren’s syndrome has shown that a reduction in Tfr cells leads to increased lymphocyte infiltration and antibody deposition in the SG (79).

### CD8^+^T cells

CD8+T cells, also known as cytotoxic T lymphocytes (CTL), are a critical subpopulation of adaptive immune cells that play a pivotal role in the elimination of intracellular pathogens. CTL are activated, proliferate, differentiate, and become effective primarily through the recognition of antigenic peptide-MHC class I molecule complexes presented by target cells via T-cells receptors (80). These cells are characterized by their ability to use perforin and MHC class I molecule complexes. CTL can kill target cells by producing IFN-γ and exhibiting cytolytic activity through perforin/granzyme or Fas cells surface death receptor (Fas) signaling pathways (81). In autoimmune diseases, the destruction of target cells by autoreactive CTL can lead to the release of large amounts of autoantigens, which may trigger the overproduction of autoantibodies. Overactive or abnormal proliferation of CTL has been observed in the SG of patients with SS (82, 83). Immunofluorescence experiments have demonstrated an increase in tissue-resident CTL within the SGECs of SS patients. CTL contribute to SG injury through multiple biological pathways. Firstly, activated CTL produce high levels of IFN-γ and TNF-α, inducing inflammatory responses in the SG (84). Secondly, IFN-γ from CTL can compromise the integrity and functionality of SGECs tight junctions, resulting in SGECs damage (85).

### B cells

In SS, the infiltrating cells in the SG are predominantly CD4^+^T cells and B cells. Initially, the infiltrating cells are mainly CD4^+^ T cells (86). Activated T cells stimulate B cells activation by producing pro-inflammatory cytokines, establishing a positive feedback loop where excessive B cells activation plays a central role in the pathogenesis of SS (87, 88). B cells contribute to the pathological process in SS by producing autoantibodies such as anti-antinuclear antibody (ANA), anti-Sjögren’s syndrome type A (anti-SSA), and anti-Sjögren’s syndrome type B (anti-SSB) antibodies (89, 90). BAFF, a member of the tumor necrosis factor superfamily, is crucial in influencing the proliferation, maturation, and survival of B cells. Produced by SG SGECs, APCs, and activated T and B cells, BAFF is a key pathogenic factor in SS. The interaction between BAFF and its receptor (BAFF-R) enhances B cells activation and proliferation, and its aberrant expression can lead to B cells dysfunction and disrupt immune homeostasis (91). Elevated BAFF levels in the SG of SS patients promote B cells maturation and proliferation, resulting in autoantibody production (88). Additionally, BAFF mediates the survival of B lymphocytes by activating the NF-κB pathway, which induces the production of B-cells lymphoma-2 (Bcl-2). Members of the Bcl-2 family can regulate SGECs apoptosis, thus supporting the survival of autoreactive B cells and the production of autoantibodies (92). Elevated BAFF levels in the serum and SG of SS patients have been found to correlate positively with autoantibody (anti-SSA/SSB) levels (93, 94). Tripartite Motif-containing 21 (TRIM21), a cytoplasmic Fc receptor, interacts with Ro60 to form Sjögren’s syndrome antigen A (SSA). TRIM21 expression enhances the binding of anti-SSA antibodies to SSA-reactive B cells, thereby driving B cells activation and anti-SSA antibody production.

Current studies have identified several novel B cells subpopulations that exert pathologic mechanisms in SS. Notable among these are abnormal B-cells subsets, such as circulating naïve B-cells and plasma cells, which are present in the SG of SS patients and positively correlate with anti-ANA antibody titers (95). T-box transcription factor TBX21(T-bet), predominantly expressed in CD4^+^ and CD8^+^T cells, regulates the development and function of Th1 cells by controlling the transcription of multiple genes. CD11c^+^ age-associated B cells (ABC) exhibit high levels of T-bet, which induces the release of IFN-γ and IL-12 (96, 97). Research has shown that an upregulation of the IL-21 signaling pathway in the SG of SS patients is associated with increased B-cells enrichment and disease activity (98), suggesting a role for CD11c^+^ ABC via IL-21 signaling (99). Fc receptor-like 4 (FcRL4) is part of the immunoglobulin superfamily and is typically expressed on human B cells, playing a regulatory role in immune responses including proliferation, differentiation, and antibody production. The proportion of FcRL4+ B cells in the SG of SS patients has been positively linked to the presence of lymphoepithelial lesions (100). FcRL4^+^ B cells are also closely associated with chemokine receptor CCR5 expression in the SG of SS patients, and their expression may enhance the migration of chemokines (CCL3 and CCL5) involved in the SG inflammatory response (101). Regulatory B cells subsets (Breg) areknown to mitigate autoimmune inflammatory responses. Bregs exert their regulatory functions through the production of regulatory cytokines and effector molecules such as IL-10, IL-35, and granzyme B (102). A negative correlation has been observed between IL-10-producing Breg cells and Tfh cells in both SS patients and SS model mice, indicating that Breg cells could represent a potential therapeutic target for SS (103).

### Dendritic cells

Dendritic cells (DC) are specialized APCs that initiate and drive the differentiation of naïve T cells into effector T cells (104). DCs can be divided into myeloid dendritic cells (mDC) and plasmacytoid dendritic cells (pDCs). In the SG of patients with SS, early infiltrating cells primarily include CD4^+^CD45RO^+^T cells and CD20^+^B cells, subsequently joined by CD27^+^ and CD79a^+^ B cells, with CD38^+^ plasma cells located at the periphery of T- and B-cells infiltrates (105). pDCs are the most potent cellular producers of type I IFN. Activation of the type I IFN pathway in the SG of SS patients correlates positively with the titers of anti-Ro and anti-La autoantibodies (106, 107). Type I IFN promotes the inflammatory response through both autocrine and paracrine mechanisms and induces BAFF production by pDCs (108). Furthermore, pDCs express TLR7 and TLR9 on their surface, which can be triggered by self-antigens to produce substantial amounts of type I IFN (109). TLR-mediated activation of pDCs leads to increased secretion of pro-inflammatory cytokines such as TNF and IL-8. Additionally, type I IFN can prompt macrophage to produce CXCL13, which in turn drives the accumulation of CXCR5^+^CD19^+^B cells in the SG, exacerbating the inflammatory response (110). mDCs on the other hand, respond primarily to microbial pathogens and facilitate Th1-mediated adaptive immune responses predominantly through the production of IL-12. It serves as a pro-inflammatory cytokine that activates T-bet to promote the differentiation of naïve T cells into Th1 cells (111). Overexpression of IL-12 in the SG of SS model mice has been observed to induce SS-like symptoms, with an age-dependent increase in anti-SSB/La and anti-ANA antibodies (112).

### Macrophage

Macrophage are among the most prevalent innate immune cells in the SG of SS patients. Studies have shown that macrophage are consistently present in the affected SG of SS patients and their abundance is positively correlated with the focality index. Activated macrophage produce inflammatory cytokines that result in damage to SGECs (113). macrophage exhibit high plasticity, differentiating into either classically activated (M1 type) or alternatively activated (M2 type) macrophage in response to various microenvironmental stimuli. M1-type macrophage exacerbate inflammation, accelerate extracellular matrix degradation and apoptosis, and enhance Th1-type immune responses. In contrast, M2 macrophage suppress T-cells proliferation and activation, modulate Th2-type immune responses, and contribute to tissue remodeling. The imbalance in M1/M2 polarization plays a critical role in the pathogenesis of SS (114, 115). M1 macrophage, prevalent in the early stages of SS, produce pro-inflammatory cytokines (TNF-α, IL-6, IL-12) that activate CD4^+^T cells, promoting their differentiation into the Th1 lineage and exacerbating SG inflammation (116). Conversely, M2 macrophage release anti-inflammatory mediators such as IL-10 and TGF-β), which help mitigate inflammation and facilitate tissue regeneration, thereby reducing autoimmune inflammation (117). As SS advances, chronic inflammation leads to irreversible SG fibrosis, primarily mediated by M2 macrophage. The TGF-β1 signaling pathway, which drives fibrosis, includes both SMAD-regulated and non-SMAD-regulated mechanisms. TGF-β1 encourages M2 macrophage polarization through activation of the SMAD2/3/4 trimeric complex, and this pathway also promotes the conversion of fibroblasts into myofibroblasts (117). Thus, the regulation of macrophage polarization homeostasis is essential in the pathogenesis of SS. In NOD/ShiLtJ mice, macrophage in the SG have been observed to produce high levels of the B-cells chemokine CXCL13, potentially facilitating the formation of ectopic GC within the SG (73).

### Salivary gland epithelial cells

The primary target organs in SS are the exocrine glands, particularly the SG. The reduction of saliva in patients is closely linked to the infiltration of immune cells around SGECs and the destruction of glandular structures (118, 119). SGECs drive and regulate local inflammatory responses by facilitating the activation and differentiation of immune cells, creating a feedback loop where immune cells and the inflammatory microenvironment further activate SGECs (120, 121). Despite not being specialized APCs, SGECs express high levels of immunoreactive molecules that mediate lymphocyte homing, antigen presentation, and amplify interactions with immune cells. Innate immune responses such as TLR signaling, inflammatory vesicle signaling, and type I interferon signaling can be activated in SGECs (122, 123). In response to inflammatory stimuli, SGECs overexpress MHC-II and co-stimulatory molecules (CD80 and CD86), which activate CD4^+^T cells in the SG (43). Additionally, SGECs highly express intercellular adhesion molecule (ICAM-1) and vascular cells adhesion molecule (VCAM-1), facilitating the binding to T-lymphocyte function-associated antigen-1 (LFA1) and very late activation antigen-4 (VLA4). This interaction stabilizes synapses between SGECs and T-cells, further mediating inflammatory responses (124). Activated SGECs also express high levels of inflammatory cytokines and chemokines (CXCL12, CXCL13, IL-1, IL-6, TNF-α and BAFF). IL-1 and IL-6 enhance APCs and CD4^+^ T-cells activation, BAFF promotes B-cells proliferation and maturation, and CXCL12 and CXCL13 facilitate the migration of predominantly B-cells immune cells to the SG (125). Growth-associated oncogene-α (GRO-α) and its receptor CXCR2, members of the CXC chemokine family, are involved in the inflammatory response in SG (126). Higher expression levels of GRO-α and CXCR2 have been observed in the SG of SS patients compared to healthy controls. Induction of SGECs with IL-6/TNF-α revealed that increased CXCR2 expression correlated positively with IL-6/TNF-α levels, and GRO-α expression was proportionally linked to CXCR2 expression under pro-inflammatory conditions (127). A disintegrin and metalloproteinase 17 (ADAM17) plays a crucial role in cells signaling, adhesion, and cytokine release, impacting inflammatory and immune responses and cells proliferation (128). A significant reduction in CXCR2 expression was observed in SS patients’ SGECs when using an ADAM17 inhibitor. It is hypothesized that ADAM17 may regulate the SG inflammatory response by modulating the interaction between GRO-α and its CXCR2 receptor (129).

Uncontrolled apoptosis can lead to the release of large amounts of cellular contents into the extracellular space, resulting in autoantigen exposure and inflammatory lesion formation, thus inducing a strong immune response. Studies have demonstrated (130) that excessively apoptotic SGECs serve as endogenous antigens, prompting pDCs to highly express IFN-α, thereby activating IFN signaling in SS patients (131). TNF-α and IFN-γ cause structural and functional disruptions in SGECs tight junctions, leading to decreased salivary secretion. Further research has shown that apoptosis in SGECs leads to the cleavage and translocation of α-cytosolic lining proteins and SSA antigens into apoptotic particles, which were then activated by pDCs, resulting in a disturbed immune response and exacerbation of the inflammatory response in the SG (132). Genetic analysis of the SG has revealed a downregulation of anti-apoptosis-related genes and upregulation of pro-apoptosis genes in SGECs in SS samples, suggesting the presence of uncontrolled apoptosis of SGECs in SS (133). Apoptosis in SGECs also activates self-reactive lymphocytes, triggering the activation of T cells, which then induce SS-associated autoantibody production and redistribution of Ro/SSA and La/SSB to the cells surface (134).

Epithelial mesenchymal transfor mation (EMT) describes the process where epithelial cells differentiate into mesenchymal cells under specific physiological and pathological conditions. TGF-β1 is a principal driver of fibrosis in many chronic inflammatory diseases (135). The activation of EMT constitutes a significant pathological response of SGECs to chronic inflammation in SS. In SS patients, fibrosis of SGECs often results from tissue injury and inflammation (136), with fibrous mediators produced by inflammatory cells and epithelial cells, particularly TGF-β1, playing crucial roles in EMT (137). SMAD proteins facilitate TGF-β1 signaling through transcriptional regulation, impacting significantly on cells function and fate (138). The snail Family Transcriptional Repressor (Snail) emerges as a pivotal regulator of EMT, reducing the connectivity and polarity of epithelial cells and facilitating their transition to mesenchymal cells (139). Sisto et al. observed a strong positive expression of EMT-related proteins (waveform protein, collagen type I, and Snail) in SG from SS patients. Conversely, the expression level of the epithelial marker E-cadherin was diminished in diseased SG biopsies, suggesting that TGF-β1 initiates the EMT program in SGECs through the classical TGF-β1/SMAD/Snail signaling pathway (140). In conclusion, SGECs play a crucial role in SS lesions. Inhibiting SGECs activation and restoring their physiological functions may enhance salivary gland secretory function in SS patients.

## AQPs and M3R are involved in regulating salivary gland secretion regulation

AquaporinS are a class of transmembrane proteins found in cells membranes, whose main role is to regulate the permeability of water molecules, thereby facilitating their passage through the cells membrane. Aquaporin 5 (AQP5) and muscarinic acetylcholine receptor subtype 3 (M3R) are integral to salivary gland secretion regulation (141, 142). AQP5, a transmembrane transporter protein, is primarily located in various secretory epithelial cells and glands such as the SG, lacrimal glands, and cornea. It enhances the cells membrane’s water permeability, facilitates water molecule transport, and participates in both secretion and absorption of water, as well as intra- and extracellular water balance (143). In coordination with AQP1 in capillary endothelial cells and myoepithelial cells, AQP5 transports saliva into the striated tubes and secretory ducts, playing a crucial role in regulating water transport rates and maintaining salivary secretion. Notably, AQP5 expression is either reduced or absent in SGECs of SS model mice, while being elevated in myoepithelial cells (144). The distribution of AQP5 is similarly diminished in the SGECs of SS patients, impeding trans-epithelial water transport within glandular vesicles and contributing to dry mouth symptoms (145). Research indicates that AQP5 expression is significantly downregulated in the SGECs of both SS model mice and SS patients (56, 146). AQP4 is found in myoepithelial cells that encircle the spinous lobules and intercalary ducts (147). In addition, it has been reported that in the SG of SS patients, there is increased AQP3 protein expression at the apical membrane of the adenohypophysis and decreased AQP1 and AQP4 protein expression in myoepithelial cellss. Therefore, as far as the current study is concerned, AQP1 and AQP3–5 may be involved in SG secretion (148).

M3R, a G-protein-coupled acetylcholine receptor, responds to acetylcholine stimulation by increasing calcium ion transport in SG alveolar cells, activating chloride channels, and mediating the translocation of AQP5 to the apical plasma membrane, thus enhancing salivary secretion (149). M3R is widely expressed in various tissues, including exocrine glands, indicating a significant role in both salivary and lacrimal secretion, particularly in the former (150). A research (151) reported that cholinergic signaling is compromised in M3R knockout mice, leading to SG hypoplasia. Furthermore, studies have shown that IL-17-producing M3R-reactive T cells may exacerbate SG inflammatory responses in SS (152). AQP5, as an effector protein of M3R, is essential for regulating salivary secretion. The anti-M3R antibody targets the M3R receptor, impairing its binding to muscarinic sites and thereby inhibiting intracellular signaling transduction, which affects AQP5 phosphorylation and its water transport capability, ultimately restricting glandular secretion (153).

## Signaling pathway

### IFN signaling pathway

Dysregulation of IFN signaling constitutes the primary pathophysiological basis of SS. Acting as a critical bridge between the innate and adaptive immune systems, IFN facilitates interactions with adaptive immune cells, perpetuating a cycle of immune activation. It modulates the immune responses of T and B cells and plays a role in SG injury (154). Enhanced IFN signaling has been observed in the SG of SS patients (155). IFNs are primarily categorized into IFN-α, IFN-β, and IFN-γ, and are activated predominantly through TLR and Retinoic Acid-Inducible Gene I-like receptor signaling (156). While most cells produce IFN-β, pDCss predominantly secrete IFN-α, linked to high expression levels of TLR7 and TLR9. IFN-α induces pDCs to express elevated levels of TLR7, resulting in a sustained increase of IFN-α in the SG of SS patients and maintaining the inflammatory milieu (157). Studies indicate that IFN-α enhances lymphocyte activation and migration to the SG via the JAK1/STAT1/2 signaling pathway in SGECs and by inducing the expression of CXCL13, BAFF, and CXCL10. Additionally, IFN-α stimulates the expression of TLR7 and the downstream signaling molecule Myeloid differentiation primary response 88 (MyD88) (158). Similarly, IFN-γ, although typically expressed at low levels in normal SGECs, is readily induced by TLR3, like IFN-α. IFN-γ also facilitates the production of autoimmune antibodies such as SSA/Ro and immunoglobulin G by stimulating BAFF expression in SGECs (159).

### TLR signaling pathway

TLRs play an integral role in the pathogenesis of SS. Current research indicates that TLRs involved in the SG inflammatory response primarily include TLR2, TLR3, TLR4, TLR7, and TLR9 (160). Moreover, viral double-stranded RNA can induce apoptosis in SGECs by activating TLR3, which upregulates apoptotic proteins such as Bcl-2 modifier and ultra-long Bcl-2 interacting cells death mediator. Apoptotic SGECs then act as endogenous antigens that recruit immune cells to infiltrate the SG, thereby triggering an immune response (161, 162). TLR2 and TLR4 are significantly upregulated in the SGs of SS mice and activate the Mitogen-Activated Protein Kinase and NF-κB pathways via MyD88, leading to elevated expression of IL-6, monocyte chemotactic protein 1, and TNF-α in SGECs. These inflammatory factors are significantly reduced when MyD88 is knocked out (163). Conversely, TLR7 and TLR9 enhance B lymphocyte maturation and proliferation. TLR9 specifically induces B lymphocytes to express autoantibodies and inflammatory factors such as monocyte chemotactic protein 1, IL-8, and IL-6. TLR7 facilitates IFN-α synthesis by inducing STAT3^S727 and NF-κB phosphorylation in B lymphocytes (164). In SS patients, TLR7 not only increases B cells secretion of IFN-α but also promotes the differentiation of immature B cells into plasma cells, culminating in abnormal immune system activation (165). Therefore, TLR signaling is critical in regulating inflammatory factor expression, B-lymphocyte maturation, antibody synthesis, and SGECs apoptosis in SS.

### BAFF/BAFF-R signaling pathway

BAFF, a member of the TNF family, induces B lymphocyte activation, maturation, migration, and autoantibody production, and is a principal cause of B lymphocyte over-activation in the SG of SS patients (166). BAFF expression is significantly higher in the SG of SS patients, correlating with serum IgG, ESR, anti-ANA levels, and disease activity. This excessive expression is a major contributor to SG damage. Studies indicate that elevated BAFF levels are present in the SG of all SS patients, while serum BAFF levels are comparatively lower in patients with GC formation than in those without (99). Consequently, the distribution of BAFF in SS patients is closely associated with lymphocyte migration to the SG and GC formation. SGECs are prompted by IFN-α to produce large amounts of BAFF, leading to the overactivation of B lymphocytes. BAFF-R, a specific receptor for BAFF, when bound to BAFF, activates the PI3K/AKT/mTOR signaling pathway in B lymphocytes. This activation leads to IKK phosphorylation and elevates the expression of NF-κB, triggering the production of inflammatory factors (IL-12, IFN-γ, IL-6, and IL-1β) and resulting in an inflammatory response in the SG (167, 168).

### JAK-STAT signaling pathway

The Janus kinase (JAK)/signal transducer and activator of transcription (STAT) pathway is a crucial regulatory system for cells proliferation and differentiation. The JAK-STAT pathway comprises four JAK intracellular tyrosine kinases (JAK1, JAK2, JAK3, and TYK2) and seven transcription factors, STAT (STAT1, STAT2, STAT3, STAT4, STAT5a, STAT5b, and STAT6), which mediate the transduction of various cytokines related to immune response, inflammation, and cellular activation and survival (169). Immunohistochemical analysis has shown that SGECs in the SG of patients with SS highly express JAK1 and JAK2 (170). Increased expression of STAT3 has been observed in SS SG (171, 172), which is stimulated by cytokines mediated by JAK1 and JAK2 (173, 174). Recent studies suggest that STAT3 is also involved in the process of episomal DNA methylation/hydroxymethylation in SS (175). In the cytokine signaling mediated by JAK/STAT, IL-6, IL-21, and IL-23 are implicated in the pathogenesis of SS. IL-21, a significant cytokine in the IFN signaling pathway, is notably elevated in SS patients, and SGRNA sequencing has revealed significantly higher levels of IL-21 and IL-21-inducible genes (IL-21R, JAK3, STAT1, and CXCL10) (176, 177). Additionally, recent studies have shown that baricitinib, a JAK inhibitor, can ameliorate the destruction of alveolar cells in the SG of SS patients by reducing IFN-γ-induced CXCL10 expression and CXCL10-dependent immune cells infiltration in the SGECs (170).

### P2X7R/NLRP3 signaling pathway

Cellular pyroptosis, a recent discovery, is a novel form of inflammatory programmed cells death characterized by mild cellular swelling. Before this swelling, bubble-like protrusions form on the cells membrane, mediated by Gasdermin family proteins. These proteins create annular pores in the membrane, allowing cellular contents to flow out gradually and triggering an inflammatory response (178). The Purinergic 2X7 receptor (P2X7R), an Adenosine triphosphate (ATP)-gated ion channel, is expressed in various tissues including the central nervous system and different epithelial tissues (179). Research has indicated (180, 181) that P2X7R in the SG plays a crucial role in cholinergic receptor-mediated salivary secretion. P2X7R is a significant activator of the NOD-like receptor family, pyrin domain-containing 3 (NLRP3) inflammasomes (182). Upon ATP stimulation, activated P2X7R facilitates the formation of large non-selective membrane pores that permit NLRP3 entry into the cytoplasm, thus activating the NLRP3 inflammasome (183). Activation of the NLRP3 inflammasome leads to the production of activated Caspase-1, which cleaves GSDMD, causing its N-terminus to perforate the cytosolic membrane. This process releases mature IL-1β and IL-18, which activate initial T-cells, prompting their differentiation into Th1 and Th17 cells and inducing the secretion of cytokines (IL-6, IL-17, IL-21, IL-22, IL-23) that provoke an inflammatory response (184). Baldini et al. demonstrated that P2X7R, Caspase-1, and IL-18 levels were elevated in the SG of SS patients at both RNA and protein levels (185). Khalafall et al. (186) showed through *in vitro* experimental studies on SS model mice that P2X7R activation prompted the assembly of NLRP3 inflammasomes and the maturation and release of IL-1β in mouse SGECs. IL-1β, a major cytokine involved in SS inflammation, primarily contributes to SGECs apoptosis and necrosis and facilitates the release of tissue-specific autoantigens. IL-18, a critical pro-inflammatory cytokine of the IL-1 family and a key downstream factor of inflammasomes, is upregulated in the SGECs of SS patients and closely linked to GC formation (187).

### TGF-β1/SMAD/Snail signaling pathway

EMT plays a crucial role in activating the pathological fibrosis cascade response in chronic inflammatory diseases (188, 189). In the pathology of SS, various cytokines significantly alter the polarity and organization of the SGECs, impacting their secretory function and closely linking to EMT activation (190, 191). Chronic inflammation is a key factor in SG fibrosis, with CD4^+^T cells, macrophage, and epithelial cells contributing to the pathological accumulation of extracellular matrix (ECM) components typical of SG changes in SS (192). Increasing evidence indicates that TGF-β1 mediates its biological effects via the TGF-β/SMAD/Snail signaling pathway, playing a significant pathogenic role in various fibrotic diseases. In SS, aberrant up-regulation of TGF-β1 in SG exacerbates the fibrosis by activating the TGF-β1/SMAD/Snail signaling pathway, leading to morphological and functional mesenchymal transformations in SGECs (63).

## Summary

The pathogenesis of SS-related salivary gland injury is complex, resulting from a multifaceted interplay of cellular and molecular factors. This process is still not fully understood, with ongoing research into its specific mechanisms. Although several potential therapeutic targets have been identified, and some targeted medications have shown promising efficacy in both ex vivo and *in vivo* studies, their translation into clinical practice remains limited, often requiring extended periods. Therefore, enhancing our understanding of SS salivary gland pathogenesis and utilizing preliminary findings to prevent or mitigate further glandular damage and preserve secretory function is essential for improving patient quality of life.

## Author contributions

WQ: Writing – original draft. JT: Funding acquisition, Methodology, Writing – review & editing. YY: Writing – review & editing. TW: Software, Writing – review & editing. YW: Formal analysis, Software, Supervision, Visualization, Writing – review & editing. ZW: Software, Writing – review & editing. GZ: Conceptualization, Writing – review & editing. YZ: Formal analysis, Software, Visualization, Writing – review & editing. JW: Conceptualization, Writing – review & editing. GW: Funding acquisition, Writing – review & editing.
